# Noninvasive Assessment of Liver Fibrosis in Patients with Chronic Hepatitis B or C by Contrast-Enhanced Magnetic Resonance Imaging

**DOI:** 10.1155/2019/3024630

**Published:** 2019-04-01

**Authors:** René Hako, Pavol Kristian, Peter Jarčuška, Ivana Haková, Ivana Hockicková, Ivan Schréter, Martin Janičko

**Affiliations:** ^1^Department of Radiology, General Hospital ZZKE, Košice 04001, Slovakia; ^2^Department of Infectology and Travel Medicine, Faculty of Medicine, University of P. J. Safarik, Košice 04001, Slovakia; ^3^1st Department of Internal Medicine, Faculty of Medicine, University of P. J. Safarik, Košice 04001, Slovakia; ^4^Department of Radiology, General Hospital, Trebišov 04001, Slovakia

## Abstract

*Background and Aim. *To develop a noninvasive magnetic resonance imaging (MRI) method for evaluation of liver fibrosis. We evaluate the utility of hepatocyte-phase Gadoxetate disodium–enhanced magnetic resonance (MR) imaging in staging hepatic fibrosis and compare it with histological analysis as the reference standard (liver biopsy).* Methods. *Prospective cohort of 78 patients, who received Gadoxetate disodium dynamic contrast-enhanced MRI (DCE-MRI), were divided into three groups. The first group (n=19) was a control group of healthy individuals without liver injury and remaining 59 subjects were chronic hepatitis B and C patients who underwent liver biopsy. These patients were divided into the mild fibrosis F1-F2 (n=32) and advanced fibrosis F3-F4 (n=27) groups. Patients were examined by generated DCE-MRI in 20th minute. Variables such as liver surface changes, homogeneities, and quantitative contrast liver/spleen ratio-Q-LSCR were evaluated and these results were consequently compared between the three groups.* Results. *Gd-EOB-DTPA contrast-enhanced dynamic liver MRI examination (DCE-MRI) can in the 20th minute differentiate mild stage of liver fibrosis (F1-F2) from severe stage of liver fibrosis (F3-F4) on the basis of liver surface changes, homogeneities, and quantitative contrast liver/spleen ratio-Q-LSCR. Diagnostic MRI criteria were created and named MRI Triple test. This test correctly identified 96% of patients with F3-F4 fibrosis and 91% of patients with the F1-F2 fibrosis in the liver biopsy. This test correctly identified 42,1% of patients in the control group (presumed F0 fibrosis without liver disease). Spearman's rank correlation coefficient (r = 0,86, P < .001) confirmed high agreement of biopsy and MR Triple test. MR Triple test's sensitivity was 96.30% (95%CI 81.03% to 99.91%), specificity 90.62% (95%CI 74.98% to 98.02%), positive predictive value 89.66% (95%CI 74.64% to 96.23%), and negative predictive value 96.67% (95%CI 80.86% to 99.50%) for discrimination between F3-4 and F1-2 fibrosis on liver biopsy.* Conclusions. *Gd-EOB-DTPA contrast-enhanced MRI liver examination in 20th minute is able to reliably differentiate mild stage of liver fibrosis (F1-F2) from severe stage fibrosis (F3-F4) on the basis of Triple test (liver surface changes, homogeneities, and quantitative contrast liver/spleen ratio-Q-LSCR).

## 1. Introduction

Liver fibrosis is caused by long-term impact of complex processes causing hepatocellular damage and triggering distorted regeneration of the liver parenchyma and accumulation of fibrous tissue. Chronic viral hepatitis (hepatitis B and C) along with chronic alcohol abuse and nonalcoholic fatty liver disease are the most common causes of liver cirrhosis; however, there are great geographical variations. Most common causes in the developed countries are alcohol misuse, and, increasingly, nonalcoholic fatty liver disease, with chronic viral hepatitis C on the decline because of effective treatment. Infection with hepatitis B virus is the most common cause in sub-Saharan Africa and most parts of Asia [1]. Possible causes further include autoimmune hepatitis, primary biliary cholangitis, primary sclerosing cholangitis, hemochromatosis, Wilson's disease, cystic fibrosis, inherited disorders of sugar metabolism (galactosemia or glycogen storage disease), or other conditions. The epidemiology of the most common causes of liver cirrhosis may vary according to the geographical location. Hepatic fibrosis is a major public health problem worldwide, which can lead to end-stage liver disease, portal hypertension, ascites, esophageal varices, and the development of hepatocellular carcinoma. Liver fibrosis is a common condition in the patients with chronic hepatitis B or C, which may progress to liver cirrhosis [2]. The early detection of hepatic fibrosis and cirrhosis has important clinical implications.

The gold standard for diagnosis and staging liver fibrosis is liver biopsy, which is invasive and expensive and carries high risk of complications. Furthermore, obtained biopsy specimens are subject to sampling variability. Inadequate biopsy sample is likely to underestimate the fibrotic stage and thus delay appropriate antiviral treatment. That is why there is a strong clinical need for noninvasive methods of identification of liver fibrosis stage. In response to the rising prevalence of chronic liver diseases, several noninvasive methods, including serum biomarkers [3], ultrasound transient elastography [4], acoustic radiation force impulse (ARFI) elastography [5], and diverse magnetic resonance (MR) imaging–based techniques, have been proposed for noninvasive diagnosis and staging of hepatic fibrosis across its entire spectrum of severity. Recent advances in magnetic resonance (MR) imaging have led to a growing interest in optimizing and applying functional MR imaging to the assessment of liver disease. Such MR imaging methods include diffusion-weighted imaging, perfusion-weighted MR imaging, MR elastography, and MR spectroscopy [2, 6].

Dynamic contrast-enhanced MR imaging (DCE-MRI) is a functional imaging using turbo gradient sequence to calculate hemodynamic and perfusion parameters in an organ. This technique is widely used in hepatocellular cancer diagnosis and it distinguishes benign and malignant tumor and monitors treatment response. Gadoxetate disodium (Gd-EOBDTPA, Primovist; Bayer Schering Pharma, Berlin, Germany), a derivative of gadopentetate dimeglumine (Gd-DTPA), is a hepatocyte-specific MR contrast agent that was developed for evaluating the hepatobiliary system. This agent is highly liver specific, and, in clinical trials, it improves the detection of focal hepatic lesions and provides diagnostic information comparable to that provided by nonspecific extracellular gadolinium chelates [6, 7]. Gd-EOBDTPA enters hepatocytes after injection and achieves maximum contrast concentration at about 10 min and 20 min. Gd-EOB-DTPA gradually secretes into bile ducts thereafter. About 50% of Gd-EOB-DTPA is excreted through the biliary system, and the other 50% is excreted by the kidney. Furthermore, hepatocyte-phase Gadoxetate disodium–enhanced MR imaging can be used for the detection or characterization of hepatic lesions and potentially for the measurement of hepatocyte function [2, 8, 9]. It is possible that DCE-MRI technique with this hepatocyte-specific contrast agent may help to diagnose and distinguish different stages of liver fibrosis. Therefore, the aim of this prospective study was to develop a noninvasive method to evaluate the severity of liver fibrosis by using comprehensive Gd-EOB-DTPA contrast-enhanced liver DCE-MRI, with histologic analysis as the reference standard.

## 2. Patients and Methods

Between December 2010 and August 2013, 69 consecutive patients with known chronic hepatitis B or C underwent liver biopsy. 10 patients were excluded from the analysis. The study exclusion criteria were inadequate biopsy specimen (n = 4) and contraindication for MRI study, such as metallic device in the body and claustrophobia (n = 3), or their MR images had severe motion artefacts due to a poor breath-holding technique (n = 3). In prospective study we finally examined and analyzed a cohort of 78 subjects (37 males 47,4%, and 41 females 52,6%, mean age, 46,0 ± 15,3 years), 59 with chronic hepatitis and 19 controls. Basic demographic data and laboratory tests (INR, albumin, platelet counts and alpha feto protein) were obtained from the patients' charts. From 59 chronic hepatitis patients (32 males 59%, 27 females 41%) 31 (39,7%) patients had chronic hepatitis B and 28 (35,9%) chronic hepatitis C with no case of coinfection.

The indication for liver biopsy in the chronic hepatitis group (*n* = 59) was the pretherapeutic assessment of liver fibrosis according to recommendations for antiviral treatment of chronic viral hepatitis, METAVIR score was used for evaluation of fibrosis.

According to histologically verified liver fibrosis using METAVIR score, 32 patients had a mild fibrosis (F1-F2) and 27 patients severe fibrosis (F3-F4). Patients with any liver tumor were excluded from the study. Control group consists of 19 healthy individuals (5 males and 14 females). These healthy controls all had normal liver function, no prior history of hepatic disease or viral hepatitis. No biopsy was performed in healthy controls.

Written informed consent was obtained from all subjects. The study was approved by Ethics Committee of Pavol Jozef Šafárik University in Košice and was performed in agreement with the Declaration of Helsinki.

### 2.1. MRI Protocol

The MRI examination procedure was adapted from the paper by Chen et al., 2012 [10]. The MR imaging was done in the 2-week interval after the liver biopsy by the same 1.5-T superconducting magnet (Magnetom Symphony; Siemens Medical Solutions, Erlangen, Germany) with a phased-array body coil. One acquisition was performed before contrast material injection, and the first contrast-enhanced acquisition started after injection of 10,0 ml (0,25 mmol/ml = 1814 mg) gadolinium-ethoxy benzyl diethylenetriamine-pentaacetic acid - Gd-EOB-DTPA (Gadoxetic acid, Primovist ®, Bayer Schering, Berlin, Germany) followed by a 20-mL saline flush injected at a rate of 2 mL/s with a MR-compatible power injector to the cubital vein via a 20-gauge intravenous catheter [10].

DCE-MRI protocol included eight consecutive transversal sequences, with a breath-hold three-dimensional T1-weighted turbo fast low angle shot sequence using the fat suppression technique (thickness/gap 8 mm/2 mm, TR 200 ms, TE 1.0 ms, flip angle 18°, FOV - field of view 42 × 29 cm; image slice thickness: 6 mm; matrix: 174 × 320, with 512 × 512 reconstruction) [10]. A total of 8x90 dynamic images were obtained for each patient. All patients were asked to breathe slowly and smoothly during imaging. Imaging of the entire liver and spleen was performed prior to (nonenhanced and contrast material–enhanced MR imaging at 25 second, 60 second, 3 min., 5 min.) and 10., 20. minutes after an intravenous bolus injection of 10 ml Gd-EOB-DTPA. After the dynamic enhancement, the static and hepatobiliary phase imaging were performed according to international consensus report. All imaging was performed by the same technician with 10 years of experience in MRI examination, to reduce possible technical errors.

### 2.2. MRI Triple Test

Postprocessing of all DCE-MRI data was performed by using a commercial software tool (Syngo 2007, Siemens Medical Solutions, Erlangen, Germany) for image segmentation and coregistration. Regions of interest (ROIs) were drawn manually as round shapes at the right hepatic segment I, V and VI in the Coinaud's liver segment classification and at the spleen. The ROIs were drawn on normal liver parenchyma away from a focal liver lesions (e.g., hepatocellular carcinoma, hemangioma, cysts, etc.), or on an abnormal bile duct dilatation, imaging artefacts, and major branches of the portal or hepatic veins. ROIs before and after contrast injection were compared at the same regions in each patient. Each liver and spleen ROIs (3 ROIs in total at the same level) were a circle (size of the ROIs ranged between 1.0 cm^2^ and 3.0 cm^2^) chosen as large as possible. These T1 signal intensity parameters (SI) were automatically calculated pixel by pixel and, finally, the medians of these parameters of pixels within drawn regions of interest were recorded for all subjects. These parameters were calculated in 20 min after contrast injection, and the diagnostic value of these parameters were compared among three different groups.

We have empirically aggregated three parameters, previously associated with high risk of fibrosis and called the resulting score MR Triple test (Table 1).

First parameter was liver-to-spleen contrast signal intensity (SI) ratio (Q-LSCR) on hepatocyte-phase images 20 minutes after contrast injection (SI_20min_). This ratio was calculated as SI_hep_ /SI_spl_, where SI_hep_ was liver signal intensity and SI_spl_ was spleen signal intensity. As we did not find guidelines on cut-off for Q-LSCR, we divided score arbitrary on three groups based on its distribution and consultation with experts. Q-LSCR ranging from 1.9 to 2.19 was considered as moderate fibrosis including also subgroup of mild fibrosis. For Q-LSCR healthy group (F0) was determined cut-off value of at least 2.2 points for moderate fibrosis (F1-F2) range values from 1.9 to 2.19 and the severe fibrosis (F3-F4) with maximum values up to 1.89.

Second parameter was the liver surface nodularity at the left liver lobe (LLS). This parameter is the most accurate marker of high stage fibrosis in ultrasound examination [11]. We have studied liver surface at the left liver lobe, and LLS was evaluated subjectively and scored on a scale from 1 to 3, with 1 corresponding to no apparent nodulations, 2 to indeterminate finding, and 3 corresponding to significant nodulation. Third parameter was liver parenchymal homogeneity (LPH), which was studied at the right and left liver lobe. LPH was also scored on a scale from 1 to 3. One point was assigned to regular homogeneous enhancement, 2 points if the enhancement was moderately inhomogeneous, and three points if severe inhomogeneous enhancement was present. Three representative MRI images at 20 minutes after contrast injection were used for LLS and LPH measurement.

The triple test result was the number of the form A + B + C (e.g., 1/1/1). Creation numbers had to be maintained in order: SI Q-LSCR 20min, liver homogeneity and surface. The resulting number was found in the decoding table with the allocation final fibrosis MRI stage (MRF0, MRF1-F2, MRF3-F4).

Imaging analysis was performed by two board certificated radiologists (10 years and 8 years of experience in abdominal DCE-MRI), who was unaware of the histologic findings. Patients were examined by generated “MR Triple test” (liver surface changes, homogeneities, and quantitative contrast liver/spleen ratio-Q-LSCR 20 minutes after contrast injection were measured on picture archiving and communication systems (PACS TomoCon, Tatramed s.r.o., Slovakia)).

### 2.3. Statistical Data Analysis

Data is expressed as means ± standard deviation (SD). The means of all the available continuous variables in the healthy and patients' groups with fibrosis were compared using the two-tailed Student's-test. The P values of signal intensity Q-LSCR parameters were tested by a one-way analysis of variance (ANOVA), used to analyze differences between F0, F1-F2, and F3-F4 groups. Scheffe's method of* post hoc *test after ANOVA was used to compare differences between the tree groups (F0 versus F1-F2, F0 versus F3-F4, and F1-F2 versus F3-F4). The Pearson's Chi-square test was used to compare three different fibrotic groups (group F0, F1-F2, and F3-F4), between biopsy and triple test in liver homogeneity and surface.

The correlation between biopsy and triple test was investigated using Pearson's, and Spearman's correlation test. The Cohen's Kappa correlation coefficient was used to compare biopsy groups F1-F2 and F3-F4 vs. Triple test groups MRF1-F2 and MRF3-F4.

The Statistical Package for Social Science Programming (version 15.0; SPSS, Chicago, IL USA) was used for analysis. A* P *value <0.05 was considered statistically significant.

## 3. Results

Patients with mild fibrosis (F1-F2 determined by histology) were significantly younger and had significantly higher levels of platelets and albumin compared to the patients with severe fibrosis and cirrhosis F3-F4 (Table 2). Regarding signal intensities of liver parenchyma Q-LSCR at 20 minutes post-Gd-EOB-DTPA injection, significant differences between F0 vs. F3-F4 and F1-F2 vs. F3-F4 were seen (P<.001). The differences between the groups F0 and F1-F2 were not significant. The highest average value of the Q-LSCR was measured in a group of F1-F2, lower in the group F0, and lowest in the F3-F4 group (Table 3). The liver homogeneity at 20 minutes post-Gd-EOB-DTPA injection correlated with fibrosis groups established by biopsy (F0, F1-F2, F3-F4), (*P*<0.001 for F0 vs. F3-F4 and F1-F2 vs. F3-F4 at 20 minutes, and* P*<0.05 for F0 vs. F1-F2) (Table 4).

**Figure 1:**
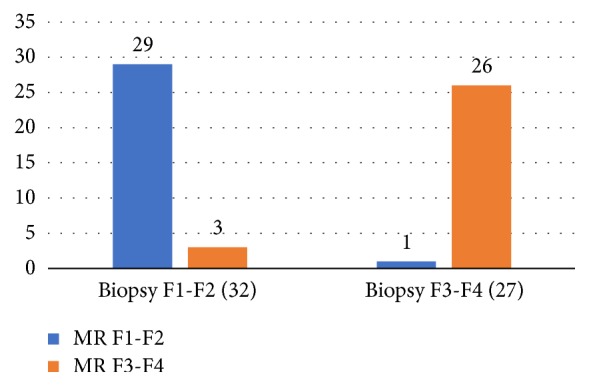
MR triple test correlation with biopsy results. Figure depicts the number of patients misdiagnosed by Triple test in two categories (significant and insignificant fibrosis).

The degree of liver surface irregularities at 20 minutes post-Gd-EOB-DTPA injection correlated with fibrosis groups established by biopsy (F0, F1-F2, F3-F4), by Pearson's Chi-square test (*P*<0.001 for F0 vs. F3-F4 and F1-F2 vs. F3-F4 at 20 minutes, and* P*<0.01 for F0 vs. F1-F2), (Table 5). In severe fibrosis MRF3-MRF4 compared to the F3-F4 group according to biopsy MRI Triple test reached 96,3% agreement in determination of the fibrosis stage. In the mild stage of liver fibrosis MRF1-MRF2 and F1-F2 according to biopsy reached 81,3% agreement and in the control sample of healthy population with presumed F0 fibrosis (without biopsy) 42,1% agreement (Figure 1). Pearson's and Spearman's rank correlation coefficient (r = 0,86, P < .001) confirmed high agreement of biopsy and MR Triple test (Table 6). Cohen's Kappa correlation coefficient for groups F1-F2 and F3-F4 according to biopsy and groups MRF1-F2 and MRF3-F4 according to MRI Triple test reached value 0,857 (P < .001). MR Triple test's sensitivity was 96.30% (95%CI 81.03% to 99.91%), specificity 90.62 % (95%CI 74.98% to 98.02%), positive predictive value 89.66% (95%CI 74.64% to 96.23%), and negative predictive value 96.67 % (95%CI 80.86% to 99.50%) for discrimination between F3-4 and F1-2 fibrosis on liver biopsy.

## 4. Discussion

Gadoxetate disodium - Gd-EOB-DTPA is a liver-specific MRI contrast agent that combines the advantages of visualizing dynamic perfusion and selective uptake by hepatocytes [12–14]. It is used most typically for accurate delineation, classification, and characterization of liver tumors [15]. After intravenous injection, Gd-EOB-DTPA is gradually taken up by the hepatocytes for up to 20 minutes. Approximately 50% of the agent is excreted via the biliary pathways and can be detected within 10 minutes after injection, the rest is excreted through the kidneys [12].

In the 1990s, a delay of 20 min for the hepatocyte phase after injection was proposed as appropriate and included in the imaging protocol for a preliminary evaluation of Gd-EOB-DTPA. Most subsequent reports have followed this protocol for [15].

Maximum liver signal intensity occurs in the hepatobiliary phase at 20 minutes after contrast injection, followed by a plateau-like enhancement lasting for about 120 minutes [8]. The hepatocyte-specific uptake of Gd-EOB-DTPA is probably an active process that includes membrane transport systems, such as OATP1 and the MRP2 [16]. The uptake of Gd-EOB-DTPA in cirrhotic livers is variable and may be difficult to predict. It is empirically well known that the enhancement of the liver is suppressed and delayed in patients with chronic liver disease during the hepatocyte phase of Gd-EOB-DTPA enhanced MR imaging [15].

Direct measurement of biliary enhancement was used to evaluate hepatic function in several studies [17–19]. The time and degree of Gd-EOB-DTPA biliary enhancement are related to hepatic function; the biliary enhancement is significantly weaker and delayed in patients with liver disease. Still, the most common approach for assessing hepatic function on Gd-EOB-DTPA-enhanced MRI is the direct or corrected measurement of hepatic parenchymal signal intensity, which is reduced in patients with hepatic dysfunction [2, 15, 20].

Decreases in hepatic enhancement on hepatocyte-phase images suggest that Gadoxetate disodium uptake by the liver is impaired, whereas the prolongation of liver enhancement suggests that Gadoxetate disodium excretion into bile is also impaired [2]. Signal intensity in the cirrhotic parenchyma tends to be lower when compared to noncirrhotic controls. Also a correlation between Child-Pugh stage and the degree of parenchymal enhancement was observed [7]. Measurements of signal intensities have also downsides significantly lowering their utility. Their high standard deviations indicate great variances and are affected by multiple confounders which are difficult to adjust for [17, 21, 22]. Lowest values of T1 relaxation times after Gd-EOB-DTPA administration were observed in patients with normal liver function and a significant increase in these times was observed in patients with liver failure (Child-Pugh C class) [7].

Possible reasons for the lower contrast enhancement in advanced fibrosis include lower number of normal hepatocytes, hepatocyte dysfunction, and fibrotic tissue accumulation that obstructs the access to the hepatocytes. Several studies have reported the evaluation of hepatic function based on the direct measurement of liver enhancement or relative enhancement (RE) measurements obtained at precontrast images (SIpre) and 20-min. postcontrast images (SIpost) as (SIpost-SIpre)/precontrast SI-pre [6, 23].

In our study, similarly to Ridge et al., we use a nondiluted standard dose of 10 mL of Gd-EOB-DTPA independent of the patient's weight; this approach works particularly well for fixed contrast regimens such as the triple arterial phase technique [24]. Relative enhancement measurements are not compatible with the use of nondiluted standard dose Gd-EOB-DTPA. The quantitative liver-spleen contrast ratio (Q-LSCR) was calculated using the signal intensities of the liver and spleen. The signal intensities of the liver and spleen were measured in 20 minutes after contrast injection. Additionally, the signal intensity and gadolinium concentration do not have a linear pattern; therefore, the signal intensity measurements may not directly correlate with the gadolinium concentration [25]. Because the intracellular uptake of Gd-EOB-DTPA decreases with impaired liver function, measurement of corrected enhancement of the contrast liver-spleen ratio (Q-LSCR) at 20 minutes after contrast injection in Gd-EOB-DTPA-enhanced images may be a new, noninvasive technique to quantify the actual function of hepatocytes.

Based on a routine clinical imaging protocol, these approaches are simple and easy to implement in clinical practice and do not require additional MR sequences, mathematical modelling, or sophisticated analysis of MR signal characteristics.

In this study, we aimed to develop the Gd-EOB-DTPA-enhanced MRI Triple test, which is a score test for liver morphology and function and compare its performance with biopsy. Our study supports the conclusion that contrast liver/spleen ratio-Q-LSCR in 20 minutes after contrast injection in Gd-EOB-DTPA-enhanced images, liver surface changes and homogeneities may be incorporated into the clinical routine as a screening test of liver imaging to detect significant liver fibrosis, without extending the acquisition time of a liver MRI protocol. This study included only patients with chronic viral hepatitis, because the etiology of cirrhosis has an impact on parenchymal changes and may thus influence the resulting Q-LSCR. Our results clearly revealed that the score based on Q-LSCR in 20 minutes, liver surface changes, and homogeneities was sufficiently able to detect mild and sever liver fibrosis [21, 26, 27].

Our study has several limitations. First, the trial was a single-centre study with a limited patient population. Due to the low number of patients we could not assess fibrosis by MRI in hepatitis B and C individually. Second, we compared the hepatic intracellular uptake of Gd-EOB-DTPA (Q-LSCR), liver surface changes and homogeneities only with the biopsy and did not evaluate liver function tests, such as indocyanine green (ICG) test, noninvasive indexes of liver fibrosis (e.g., APRI, FIB-4) or morphological tests such as elastography. Therefore, further studies are required in this respect. Third, the MRI images were evaluated by two different radiologists and of each patient was evaluated by only one radiologist. Fourth, sampling variation of biopsy may exist. Also considering the recent advances in rapid MR imaging, radiologists and radiological technologists find it stressful to wait for 20 min for the hepatocyte phase [15].

## 5. Conclusion

Gadoxetate disodium dynamic contrast-enhanced MRI is a noninvasive and quantitative method to evaluate liver functional status and liver morphology. The hepatospecific phases provide useful information about liver fibrosis and can be easily incorporated into to clinical practice, without additional financial cost. MRI Triple test may prove be suitable and robust for detecting and characterizing liver fibrosis. Additionally, this method may be useful also for monitoring disease progression.

## Figures and Tables

**Table 1:** Triple test scoring system.

	1 point	2 points	3 points

Q-LSCR 20min Signal intensity	*> 2,2*	*1,9 – 2,19*	*< 1,89*

Homogeneity grade	regular homogeneous enhancement	moderately inhomogeneous enhancement	severe inhomogeneous enhancement

Surface	no apparent nodulations	indeterminate	significant nodulation

Q-LSCR: quantitative contrast liver/spleen ratio.

**Table 2:** Baseline parameters of the study cohort.

	Biopsy	n	min.-max. (range)	mean (STD)	Sig. (P) (t-test)	t-test df
Age(years)	F1-F2	32	23-60	36,41(9,55)	-8,145*∗∗∗*	57
	F3-F4	27	35-78	58,44(11,24)		F1+F2<F3+F4

Weight(kg)	F1-F2	32	35-108	73,06(18,34)	0,124 ^NS^	57
	F3-F4	27	42-120	72,48(17,46)		F1+F2>F3+F4

Height(cm)	F1-F2	32	152-197	171,87(11,26)	3,015*∗∗*	57
	F3-F4	27	153-182	163,93(8,48)		F1+F2>F3+F4

BMI(kg/m^2^)	F1-F2	32	15,1-38,6	24,57(5,29)	-1,617 ^NS^	57
	F3-F4	27	16,4-39,2	26,79(5,23)		F1+F2<F3+F4

Platelets	F1-F2	32	147-341	227,75(61,24)	4,188*∗∗∗*	57
(x10^9^/L)	F3-F4	27	36-294	157,74(67,09)		F1+F2>F3+F4

Albumin	F1-F2	32	42,3-51,1	47,18(2,2)	4,358*∗∗∗*	30,117
(g/L)	F3-F4	27	27,3-52,2	40,92(7,19)		F1+F2>F3+F4

INR	F1-F2	32	0,9-1,4	1,046(0,1)	-3,29*∗∗*	33,715
	F3-F4	27	0,9-2,0	1,209(0,24)		F1+F2<F3+F4

AFP	F1-F2	32	0,77-17,91	3,64(3,31)	-3,436*∗∗*	34,155
(kIU/L)	F3-F4	27	0,92-34,91	9,08(7,65)		F1+F2<F3+F4

*∗∗∗*p<0,001, *∗∗*p<0,01, *∗*p<0,05, NS: nonsignificant.

**Table 3:** The liver signal intensity Q-LSCR parameter differences at 20 minutes post-Gd-EOB-DTPA injection among different categories of fibrosis.

	Biopsy	N	min.-max.	mean (STD)	Group comparison sig., F (ANOVA)	Post hoc Sheffe, sig. (P)
Q-LSCR	F0	19	1,31-3,01	2,19(0,39)	*∗∗∗*15,38	F0<F1+F2 ^NS^
20 min.	F1-F2	32	0,99-2,98	2,24(0,39)		F0>F3+F4*∗∗∗*
	F3-F4	27	1,27-2,71	1,77(0,33)		F1+F2>F3+F4*∗∗∗*

Q-LSCR = quantitative liver-spleen contrast ratio; differences between F0, F1-F2, and F3-F4 biopsy groups were analyzed by ANOVA; Scheffe's method of *post-hoc *test was used to compare differences between the tree groups (F0 versus F1-F2, F0 versus F3-F4, and F1-F2 versus F3-F4); *∗∗∗*p<0,001, *∗∗*p<0,01, *∗*p<0,05, NS: nonsignificant.

**Table 4:** The differences of liver homogeneity at 20 minutes post-Gd-EOB-DTPA injection among different categories of fibrosis.

		Biopsy n (%)				
MRI homogeneity grade		F0	F1-F2	F3-F4	Comparison	Chi-squared (Pearson), sig. (P),
Homogeneity	1	9(47,4)	4(12,5)	0(0,0)	F0 vs. F1+F2	*∗*8,59
20 min.	2	10(52,6)	25(78,1)	7(25,9)	F0 vs. F3+F4	*∗∗∗*29,02
	3	0(0,0)	3(9,4)	20(74,1)	F1+F2 vs. F3+F4	*∗∗∗*26,45

Differences between F0, F1-F2, and F3-F4 biopsy groups were analyzed by Pearson's chi-square test; *∗∗∗*p<0,001, *∗∗*p<0,01, *∗*p<0,05, NS: nonsignificant.

**Table 5:** The differences of liver surface parameter at 20 minutes post-Gd-EOB-DTPA injection among different categories of fibrosis.

MRI liver		biopsy n (%)				
surface irregularity grade		F0	F1-F2	F3-F4	Comparison	Chi-squared (Pearson), sig. (p),
Liver surface	1	14(73,7)	10(31,3)	0(0.0)	F0 vs. F1+F2	*∗∗*8,62
20 min.	2	5 (26,3)	22(68,8)	3(11,1)	F0 vs. F3+F4	*∗∗∗*38,27
	3	0(0,0)	0(0,0)	24(88,9)	F1+F2 vs. F3+F4	*∗∗∗*48,36

Differences between F0, F1-F2, and F3-F4 biopsy groups were analyzed by Pearson's chi-square test; *∗∗∗*p<0,001, *∗∗*p<0,01, *∗*p<0,05, NS: nonsignificant.

**Table 6:** The correlation between biopsy and Triple test.

Groups		MR Triple test prediction n (%)				
		MR F0	MR F1-F2	MR F3-F4	Pearson's correlation coefficient sig. (P),	Spearman's correlation test coefficient (r), sig. (P),
Biopsy	F0 (presumed)	8(42,1)	10(52,6)	1(5,3)	0,86*∗∗*	0,86*∗∗*
	F1-F2	3(9,4)	26(81,3)	3(9,4)	(0,000)	(0,000)
	F3-F4	0(0,0)	1(3,7)	26(96,3)		

Pearson's and Spearman's correlation tests were used to compare biopsy groups (F0, F1-F2, and F3-F4) vs. Triple test groups MRF0, MRF1-F2, and MRF3-F4.

Correlation *∗∗*p<0,01, NS: nonsignificant, sig. *∗∗∗*p<0,001, *∗∗*p<0,01, *∗*p<0,05, NS: nonsignificant.

## Data Availability

Dataset is not available. Due to GDPR EU regulation, we cannot publish individual, although anonymized, patient data unless we are given explicit consent to publish these data from each participant. Since the recruitment of the patients was from 2010 to 2013, informed consent signed at the beginning of the study did not contain the provision to publish individual patient data; we are legally forbidden to publish the dataset.
